# Fructose promotes pyoluteorin biosynthesis via the CbrAB-CrcZ-Hfq/Crc pathway in the biocontrol strain *Pseudomonas* PA1201

**DOI:** 10.1016/j.synbio.2023.09.004

**Published:** 2023-09-21

**Authors:** Ying Cui, Kai Song, Zi-Jing Jin, Learn-Han Lee, Chitti Thawai, Ya-Wen He

**Affiliations:** a State Key Laboratory of Microbial Metabolism, Joint International Research Laboratory of Metabolic and Developmental Sciences, School of Life Sciences and Biotechnology, Shanghai Jiao Tong University, Shanghai, 200240, China; b Novel Bacteria and Drug Discovery Research Group (NBDD), Microbiome and Bioresource Research Strength (MBRS), Jeffrey Cheah School of Medicine and Health Sciences, Monash University Malaysia, Bandar Sunway, Selangor Darul Ehsan, 47500, Malaysia; c Department of Biology, Faculty of Science, King Mongkut's Institute of Technology Ladkrabang, Bangkok, 10520, Thailand

**Keywords:** *Pseudomonas*, Biocontrol, Pyoluteorin, Fructose, Carbon catabolism repression

## Abstract

Biocontrol strain *Pseudomonas* PA1201 produces pyoluteorin (Plt), which is an antimicrobial secondary metabolite. Plt represents a promising candidate pesticide due to its broad-spectrum antifungal and antibacterial activity. Although PA1201 contains a complete genetic cluster for Plt biosynthesis, it fails to produce detectable level of Plt when grown in media typically used for *Pseudomonas* strains. In this study, minimum medium (MM) was found to favor Plt biosynthesis. Using the medium M, which contains all the salts of MM medium except for mannitol, as a basal medium, we compared 10 carbon sources for their ability to promote Plt biosynthesis. Fructose, mannitol, and glycerol promoted Plt biosynthesis, with fructose being the most effective carbon source. Glucose or succinic acid had no significant effect on Plt biosynthesis, but effectively antagonized fructose-dependent synthesis of Plt. Promoter-*lacZ* fusion reporter strains demonstrated that fructose acted through activation of the *pltLABCDEFG* (*pltL*) operon but had no effect on other genes of *plt* gene cluster; glucose or succinic acid antagonized fructose-dependent *pltL* induction. Mechanistically, fructose-mediated Plt synthesis involved carbon catabolism repression. The two-component system CbrA/CbrB and small RNA catabolite repression control Z (crcZ) were essential for fructose-induced Plt synthesis. The small RNA binding protein Hfq and Crc negatively regulated fructose-induced Plt. Taken together, this study provides a new model of fructose-dependent Plt production in PA1201 that can help improve Plt yield by biosynthetic approaches.

## 1 Introduction

Pyoluteorin (Plt) is an aromatic polyketide metabolite produced by diverse *Pseudomonas* strains and composed of a resorcinol ring and a dichloropyrrole [[1], [2], [3], [4]]. Plt is best known for its toxicity against *Pythium ultimum*, an important soil-borne plant pathogen that causes damping-off of over 300 diverse plant species, including cucumber and other cucurbits [[3], [4], [5]]. Plt also inhibits bacteria and fungi that impact on human health or crop production such as *Mycobacterium tuberculosis hominis* and *Phytophthora infestans*, respectively [2]. More recently, Plt was demonstrated to inhibit the fungal forest pathogen *Heterobasidion* spp, which causes destructive root and butt rots in coniferous forests of the Northern Hemisphere [6]. The presence of one or more electron-withdrawing groups on Plt's pyrrole is required for its antibacterial activity [7]. In parallel to these antibiotic properties, Plt has become a lead candidate compound for drug discovery against human triple-negative breast cancer and non-small cell lung cancer [8,9]. Thus, since the 1980's, Plt biosynthesis has attracted researchers' attention.

*Pseudomonas aeruginosa* M18, and *P. protegens* Pf-5 and H78 are three well-studied Plt-producers [[10], [11], [12]]. In *P. protegens* Pf-5, Plt production is associated with the gene cluster *pltMRLABCDEFGZHJKNO* [11]. *PltLABCDEFG* encodes the enzymes responsible for polyketide synthesis (PKS) and non-ribosomal peptide synthesis (NRPS), two components essentials for Plt synthesis [13], while the *pltHIJKNO* operon encodes an ATP-binding cassette (ABC) transporter thought to be involved in Plt efflux [14]. Moreover, Plt synthesis involves two transcription factors: PltR and PltZ [11,13]. PltM was elegantly demonstrated to catalyze the mono- and dichlorination of phloroglucinol, a compound that serves as potent transcriptional regulator of Plt biosynthesis, without the need for a biosynthetic intermediate [15].

In *P*. *aeruginosa*, Plt biosynthesis is strictly regulated by a complex protein network. PltR, a LysR family regulator, binds *pltL* promoter to activate *pltL* expression [10]. The TetR family regulator PltZ recognizes a semi-palindromic sequence in the promoter region of the *pltHIJKNO* operon [16]. While PltR is required for Plt autoinduction, it is not sufficient, and the direct binding of PltZ to Plt should concur [11]. In addition, Plt biosynthesis is regulated by a range of pathways, such as the Gac/Rsm network and quorum sensing (QS) systems [[17], [18], [19], [20], [21]].

Compared to intrinsic or QS regulation, the effects of environmental cues and medium nutrients on Plt production are relatively less studied. Decent amount of research has been done to understand the impact of gluconic acid, mannitol and glycerol on the Plt production of *Pseudomonas* CHA0 [22,23]. Additionally, Plt production of *Pseudomonas* S272 is known to be repressed by glucose and favorited by ethanol and glycerol [24].

Carbon catabolism repression (CCR) is a main regulator of bacterial growth and metabolite biosynthesis [25]. It is a general mechanism that governs the sequential use of carbon sources in microbes. This mechanism promotes the use of nutrients that support high growth rates and consequently limits the expression of genes that are not essential for growth, preventing high metabolic cost [26]. In *P. aeruginosa*, there is a hierarchy of substratum preferences. It begins with the use of amino acids such as aspartate, followed by citrate, succinate, lactate, acetate, and ultimately glucose [26]. The CCR-related protein catabolite repression control (Crc), and the small RNA (sRNA) binding protein Hfq are two key components involved in the CCR-dependent regulation [27]. Crc-bound Hfq binds to the A-rich motifs on target mRNA near to ribosome binding site, thereby preventing their translation [28]. The transcription of regulatory sRNAs, including *CrcZ*, is activated by the two-component signaling system CbrA/CbrB [29]. Thus, Hfq binds to *CrcZ* and Crc protein to form a regulatory complex [30,31].

PA1201 is a *P. aeruginosa* strain that was originally isolated from the rice rhizosphere, and was shown to display strong inhibitory activity towards the pathogens *Rhizoctonia solani* and *Xanthomonas oryzae* pv. *oryzae* [32]. PA1201 contains a Plt biosynthetic gene cluster which is highly homologous to those identified in other *Pseudomonas* strains such as Pf-5 and M18. However, PA1201 failed to produce any detectable level of Plt during growth at 28 °C in KMB medium [32].

In this study, we tested whether the carbon source in the growth medium and CCR influence Plt synthesis. Such clues may serve to improve Plt yield via biosynthesis in the biocontrol strain *Pseudomonas* PA1201.

## 2 Materials and methods

### 2.1 Bacterial strains, media, and growth conditions

The bacterial strains and plasmids used in this study are described in Tables S1 and S2. *Escherichia coli* strains were grown aerobically at 37 °C in Luria-Bertani (LB) [33]. When required, 20 μg/mL 5-bromo-4-chloro-3-indolyl-β-d-galactopyranoside (X-Gal) was used for blue/white colony screening. The following media with difference carbon sources were used for PA1201 culture: KMB [34]; PPM [35]; LB; MM [36]; M (MM medium without mannitol). All the strains were grown at 28 °C in Erlenmeyer flasks (250 mL) at 220 rpm in a rotary shaker (ZQWY-200 N, SH Zhichu, China). Antibiotics were added at the following concentrations when needed: 100 μg/mL spectinomycin (Spe); 50 μg/mL kanamycin (Kan); and 20 μg/mL tetracycline (Tet). All chemicals were purchased from Sangon Biotech (Shanghai).

### 2.2 Quantitative analysis of Plt level in PA1201 cultures

A total of 500 μL of the appropriate culture was collected and extracted with 1 mL of ethyl acetate. The organic phase was subsequently collected and evaporated. The residues were dissolved in 100 μL of methanol for analysis by HPLC (Agilent Technologies 1260 Infinity). A 5-μL sample was injected into a C18 reverse-phase column (Zorbax XDB; 5 μm, 4.6 × 150 mm) with a flow rate of 1 mL/min with the following steps: solvent A was water plus 0.1% (vol/vol) acetic acid, while solvent B was acetonitrile plus 0.1% (vol/vol) acetic acid. The column was preequilibrated in 90% solvent A–10% solvent B and was eluted using a linear gradient. After separation of an injected sample, the column was equilibrated in 90% solvent A–10% solvent B for 4.9 min prior to the next injection. Under these chromatographic conditions, Plt was eluted at 11.05 min. Quantification was performed by integrating the peak area under the wavelength at 300 nm and Plt concentration using the standard curve obtained with a commercial Plt. Due to the different growth rate of PA1201 strains in different media, Plt level was defined as mg/(OD_600_.L) to normalize Plt production of the same population.

### 2.3 Construction of *lacZ*-dependent reporter strains for transcriptional assay

The method for constructing promoter-*lacZ* fusion reporter strains in PA1201 was previously described by Becher and Schweizer [37]. Briefly, the promoter region of a target gene (approximately 500 bp upstream of the start codon) was amplified by PCR. The primers used for the different reporter strains are listed in Table S3. The PCR products were then cloned into the vector mini-CTX-lacZ. The recombinant plasmids were integrated into the chromosomes of the PA1201-derived strains at the *attP* site. The β-galactosidase activity was measured as previously described [38].

### 2.4 Gene deletion and functional complementation analysis

The method used for in-frame gene deletion was previously described elsewhere [35]. Briefly, the upstream and downstream regions of the gene to be deleted were fused by overlap extension PCR. The fusion product was then subcloned into the suicide vector pK18mobsacB carrying the sucrose-sensitive *sacB* gene. The resulting recombinant plasmid was introduced into PA1201 through mating, and the plasmid was subsequently integrated within the target gene by homologous recombination. The resulting strain was then plated on LB agar plate with 50 μg/mL Spectinomycin (Spe) and 5% (w/v) sucrose for a second single crossover homologous recombination event, resulting in allelic exchange. The resulting mutant was verified by PCR and subsequent DNA sequencing. The primers used for the PCR and subsequent screening are listed in Table S3.

For complementation analysis, the target gene was amplified by PCR and cloned into the PBBR-1-MCS plasmid. The different constructs were then transferred into PA1201 through triparental mating. Triparental mating between PA1201 and *E. coli* was carried out with the helper strain *E. coli* (pRK2013). The primers used for this process are shown in Table S3.

### 2.5 Statistical analysis

All experiments were performed at least in triplicate independently. The ANOVA tests for all experimental datasets were performed using the JMP software program (version 5.0). The significant effects of the different treatments were assessed by F values. The differences with significant F tests underwent further analysis by separation of means with Fisher's protected least significant difference test using *p* < 0.05.

## 3 Results

### 3.1 Nutrient-poor MM medium favors Plt production in PA1201

**Fig. 1:**
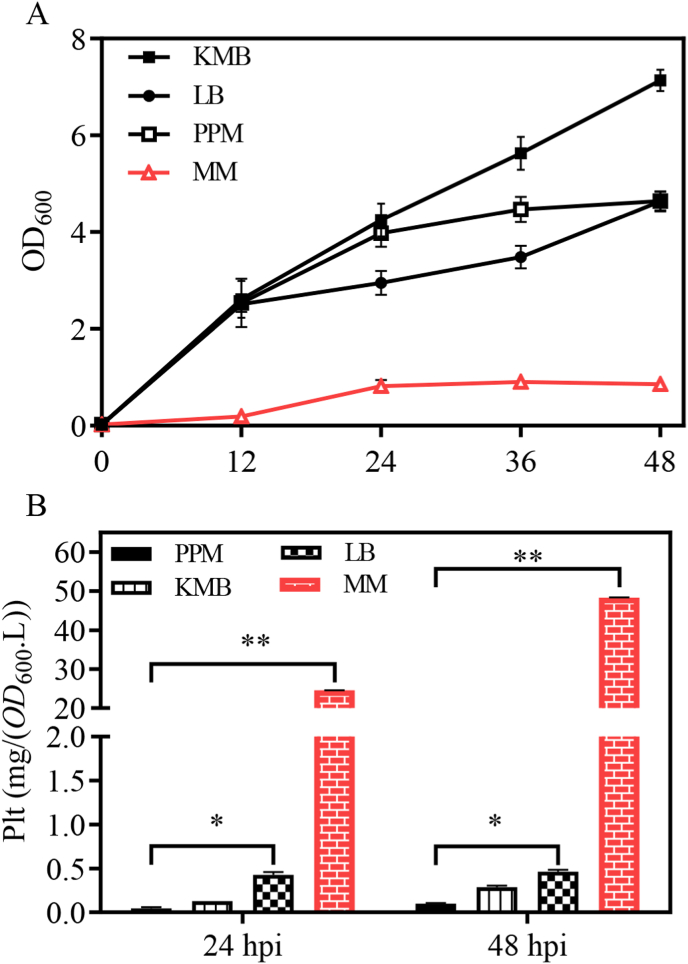
**Plt production by PA1201 strain in different media.** (A) PA1201 growing rate in KMB, LB, PPM and MM media. (B) Plt production at 24- and 48-h post inoculation (hpi).

First, we compared the effect of different media on Plt production by *Pseudomonas* PA1201. PA1201 was inoculated to and grown in four types of media, *i.e.*, KMB, LB, MM, and PPM for 48 h at 28 °C. At the endpoint, Plt in the different cultures was quantified by HPLC, using a commercially available Plt sample as reference (Fig. S1). PA1201 cultures grew best in KMB, LB, and PPM media, reaching OD_600_ ranging from 7.3, 4.8 and 4.7, whereas MM medium supported PA1201 growth poorly, with an OD_600_ of 0.9 at 48 h post inoculation (hpi) (Fig. 1A). However, MM medium yielded the highest concentration of Plt, with 24.5 mg/(OD_600_.L) at 24 hpi and 48.3 mg/(OD_600_.L) at 48 hpi; Plt concentration in KMB, LB, and PPM medium were less than 0.5 mg/(OD_600_.L) (Fig. 1B).

MM is a nutrient-poor medium with mannitol as the major carbon source. To further determine the effect of medium composition on Plt production, two media, 1/3 KMB medium, containing one third of all KMB components, and KMBM, containing all KMB components supplemented with 10 g/L mannitol, were prepared. No improvement in Plt yield was observed in 1/3 KMB or KMBM (Fig. S2), suggesting that Plt biosynthesis in MM was not improved by nutrient limitation or the unique availability of mannitol as carbon source, but rather, it was involved other specific regulators.

### 3.2 Fructose is the optimal carbon source for Plt biosynthesis

**Fig. 2:**
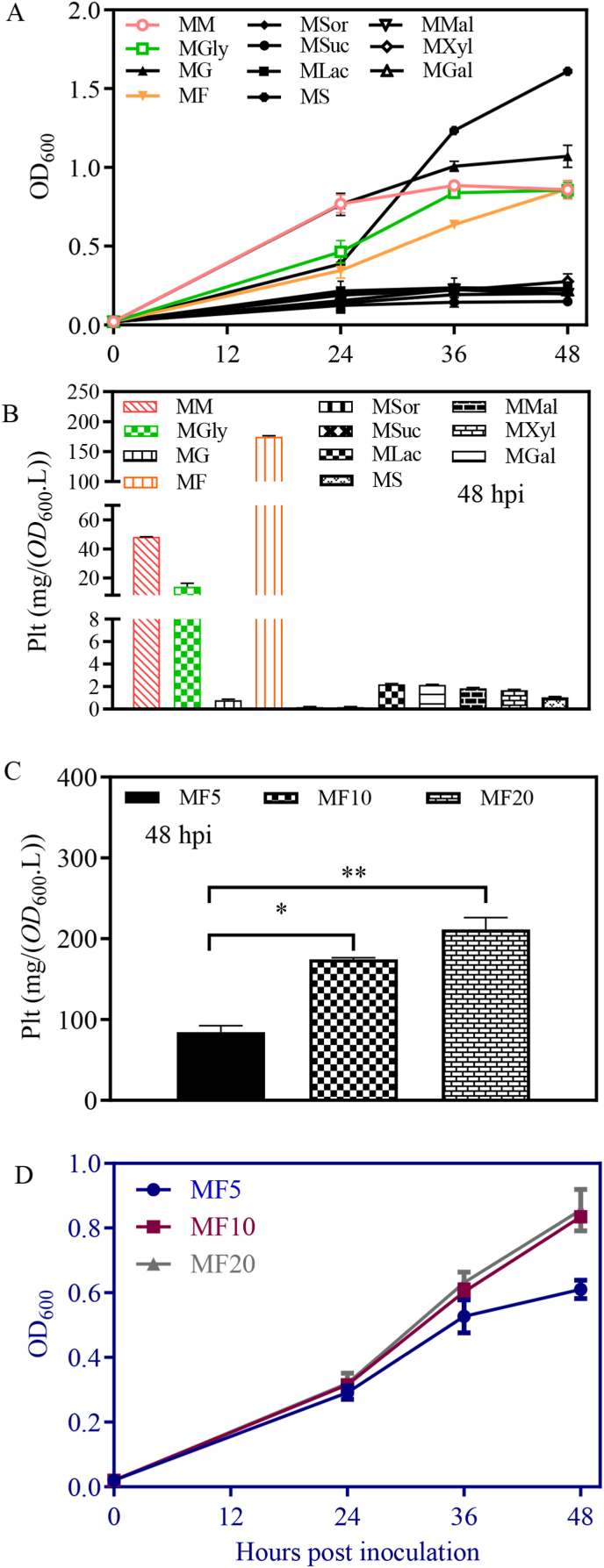
**Fructose promotes Plt production in minimal medium (M).** (A) Growth curve of PA1201 in minimal M medium (MM medium without mannitol), supplemented with 5 mM carbohydrates, as indicated: Fru: fructose; Mal: maltose; Glu: glucose; Sor: sorbitol; Suc: sucrose; Lac: lactose; Gal: galactose; Xyl: xylose; Succ: succinic acid. (B) Plt level in PA1201. (C) Plt level at 48 hpi in M medium supplemented with 5–20 mM fructose. (D) Growth curve of PA1201 in M medium supplemented with 5–20 mM fructose.

Carbon sources are key to bacterial growth and metabolite production. To determine the effects of different carbohydrates on PA1201 growth and Plt biosynthesis, a M medium with the same composition as MM medium except for mannitol was used as basis. Mannitol, glycerol, glucose, fructose, sorbitol, galactose, sucrose, lactose, maltose, xylose, and succinic acid was respectively added to M medium at a final concentration of 10 mM to generate the media MM, MGly, MG, MF, MSor, MGal, MSuc, MLac, MMal, MXyl, and MS. Succinic acid and glucose significantly increased PA1201 growth (Fig. 2A), with succinic acid being the most effective. Regardless, high level of Plt was only observed with mannitol, fructose, or glycerol supplementation (Fig. 2B), with fructose being the most effective carbon source, reaching 174.6 mg/(OD_600_.L) at 48 hpi at 10 mM and displaying a dose-dependent effect at concentrations ranging from 5 mM to 20 mM (Fig. 2C).

### 3.3 Glucose or succinic acid antagonizes fructose-promoted Plt biosynthesis

**Fig. 3:**
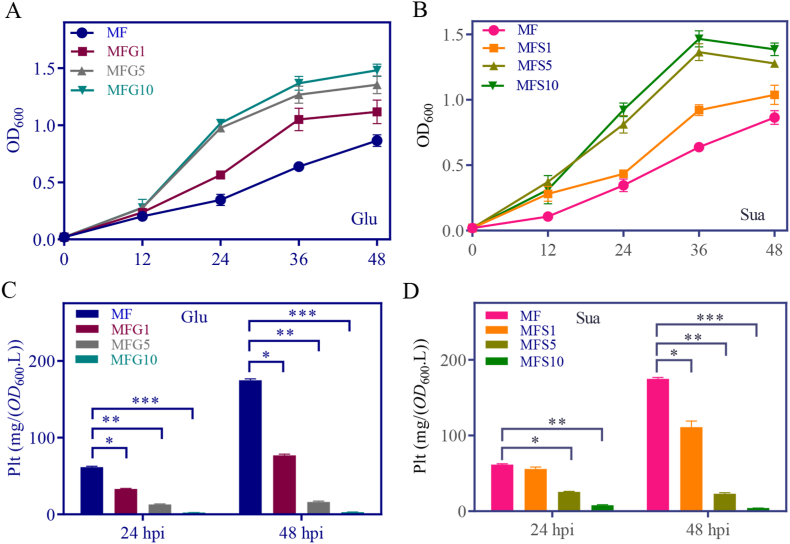
**Glucose or succinic acid antagonizes fructose-induced Plt biosynthesis.** (A) PA1201 growth over time in MF medium supplemented with 1–10 mM glucose, or (B), 1–10 mM succinic acid. (C) Plt levels at 24- and 48-hpi in MF medium supplemented with 1–10 mM glucose, or (D), 1–10 mM succinic acid.

In keeping with a previous report [39], we found that glucose and succinic acid were the preferred carbon sources for *Pseudomonas* PA1201 growth (Fig. 2A). To attempt combining the growth-promoting effect of glucose or succinic acid with the Plt-promoting effect of fructose, and further improve Plt yield, glucose or succinic acid was respectively added into the MF medium at final concentrations of 1, 5, and 10 mM, which generated respectively, the media MFG1, MFG5, MFG10, MFS1, MFS5, and MFS10. Addition of glucose or succinic acid to MF medium significantly promoted PA1201 growth (Fig. 3A and B) but decreased Plt levels in a dose-dependent manner (Fig. 3C and D). These results suggested an antagonistic effect between fructose and glucose, or fructose and succinic acid for Plt biosynthesis.

### 3.4 Both the promoting effect of fructose and the antagonizing effect of glucose or succinic acid on Plt biosynthesis are mediated by the operon *pltL*

**Fig. 4:**
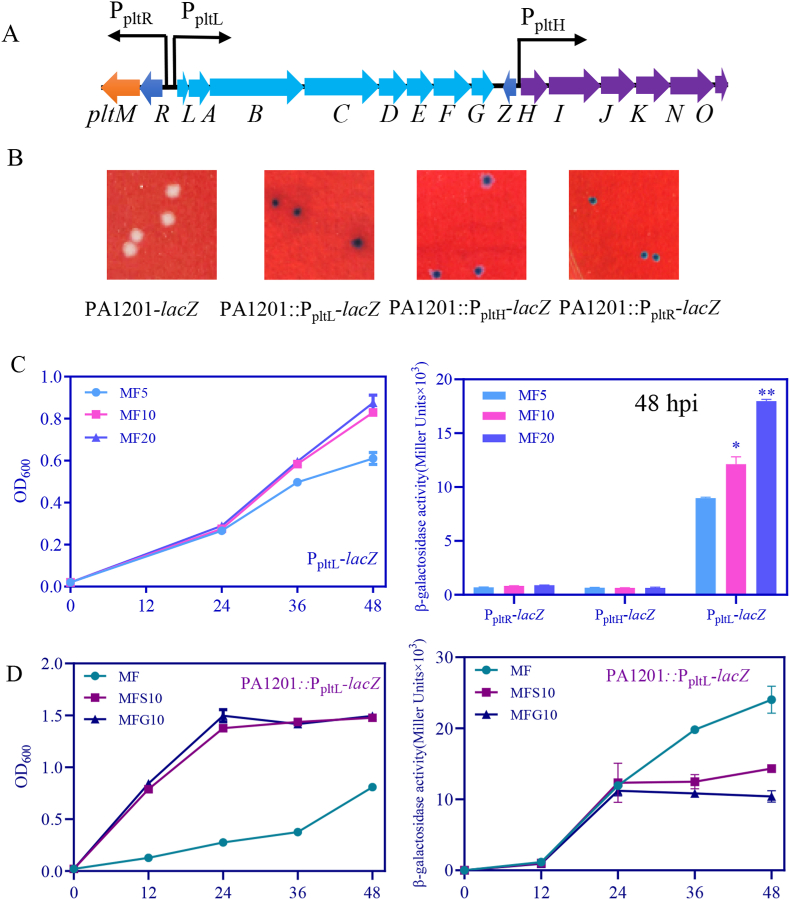
**Effects of fructose, glucose, and succinic acid on *pltL* expression.** (A) Plt gene cluster and the three studied promoters. (B) Representative pictures showing the colonies carrying different reporter transgenes PA1201-*lacZ* (negative control), PA1201:P_pltL_-*lacZ*, PA1201:P_pltH_-*lacZ* and PA1201:P_pltR_-*lacZ* on the MF agar plate supplemented with 40 mg/L X-gal. (C) Effects of 5–20 mM fructose on P_pltR_-, P_pltH_- and P_pltL_-dependent β-galactosidase activity in PA1201 at 48 hpi. (D) P_pltL_-dependent β-galactosidase activity in PA1201 cultured in MF, MFS10, and MFG10 media.

In PA1201, Plt biosynthesis relies on the gene cluster *pltMRLABCDEFGHIJKNO*, composed of at least three operons, *i.e.*, *pltR*, *pltL* and *pltH* (Fig. 4A). To monitor the activity of these operons upon exposure to different carbon sources, three reporter strains, PA1201:P_pltL_-*lacZ*, PA1201:P_pltH_-*lacZ*, and PA1201:P_pltR_-*lacZ*, were generated as previously described [37]. In MF agar plates supplemented with X-gal, PA1201:P_pltH_-*lacZ* and PA1201:P_pltR_-*lacZ* colonies exhibited a light blue color, while the PA1201:P_pltL_-*lacZ* colonies exhibited a dark blue color, provoked by the degradation of X-gal substrate by the reporter enzyme β-galactosidase, encoded by *lacZ* under the control of *PltL* (Fig. 4B). The quantification of the β-galactosidase activity confirmed that the promoter P_pltL_ was activated to a higher level than P_pltH_ or P_pltR_ in presence of 10 mM fructose (Fig. 4C). Fructose upregulated P_pltL_ activity in a dose-dependent manner (Fig. 4C), while increasing fructose concentration did not modify P_pltR_ or P_pltH_ activity (Fig. 4C). These results suggested that the effect of fructose on Plt production is mediated by the *pltL* operon.When 10 mM glucose (MFG10) or 10 mM succinic acid (MFS10) were added to MF liquid medium, P_pltL_ activity was significantly lowered compared with that observed on MF liquid medium (Fig. 4D), suggesting that glucose or succinic acid antagonized the effect of fructose on operon *pltL,* and consequently, on Plt biosynthesis.

### 3.5 The two-component signal system CbrA/CbrB is essential for fructose-dependent induction of Plt biosynthesis

**Fig. 5:**
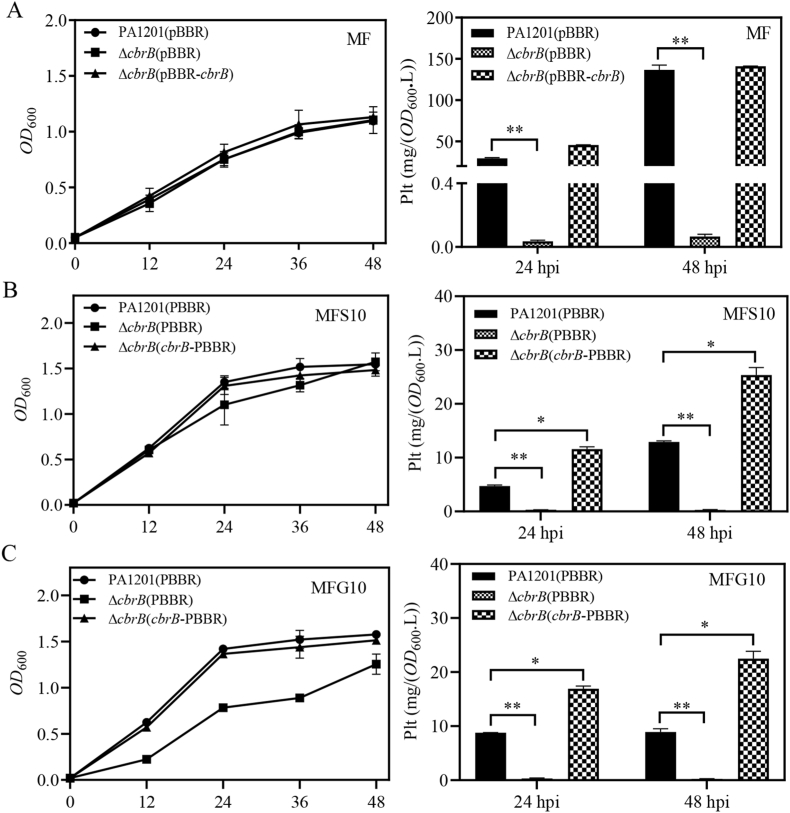
**CbrB positively regulates Plt biosynthesis in PA1201.** (A) Growth time course and Plt production in the strains PA1201(pBBR), Δ*cbrB*(pBBR) and Δ*cbrB*(pBBR-*cbrB*) at 24- and 48-hpi in MF medium, in (B) MFS medium, and in (C) MFG medium.

The CbrA/CbrB system is unique to bacteria of the *Pseudomonaceae* family. It integrates various signals and regulates multiple physiological processes involved in bacterial adaptation to varying environments [40]. To investigate the possible role of CbrA/CbrB in fructose-dependent Plt induction, strains either deleted for *cbrB* [Δ*cbrB*(pBBR)] or deleted and complemented with overexpressed CbrB [Δ*cbrB*(pBBR-*cbrB*)] were generated and cultured in MF medium. These genetic alterations did not alter PA1201 growth in MF medium (Fig. 5A). Nonetheless, mutation *cbrB* markedly reduced the Plt production at 24 or 48 hpi, whereas *cbrB* overexpression restored Plt expression to wild-type level in the Δ*cbrB*(pBBR-*cbrB*) strain (Fig. 5A). These results suggested that the CbrA/CbrB system is required for fructose-promoted Plt biosynthesis.

Similarly, in the MFS10 or MFG10 medium, containing respectively the antagonist glucose or succinic acid, Plt synthesis was not detectable with Δ*cbrB*(pBBR), but was restored beyond wild-type level with Δ*cbrB*(pBBR-*cbrB*)*,* albeit Plt levels in these media remained below those in MF for all strains (Fig. 5B and C). These observations suggested that the inhibition exerted by glucose or succinic acid was slightly overcome by CbrB overexpression, implying that CbrA/CbrB may participate to the antagonization of fructose-promoted Plt biosynthesis by these nutrients.

### 3.6 The sRNA *CrcZ* is essential for fructose-dependent induction of Plt biosynthesis

**Fig. 6:**
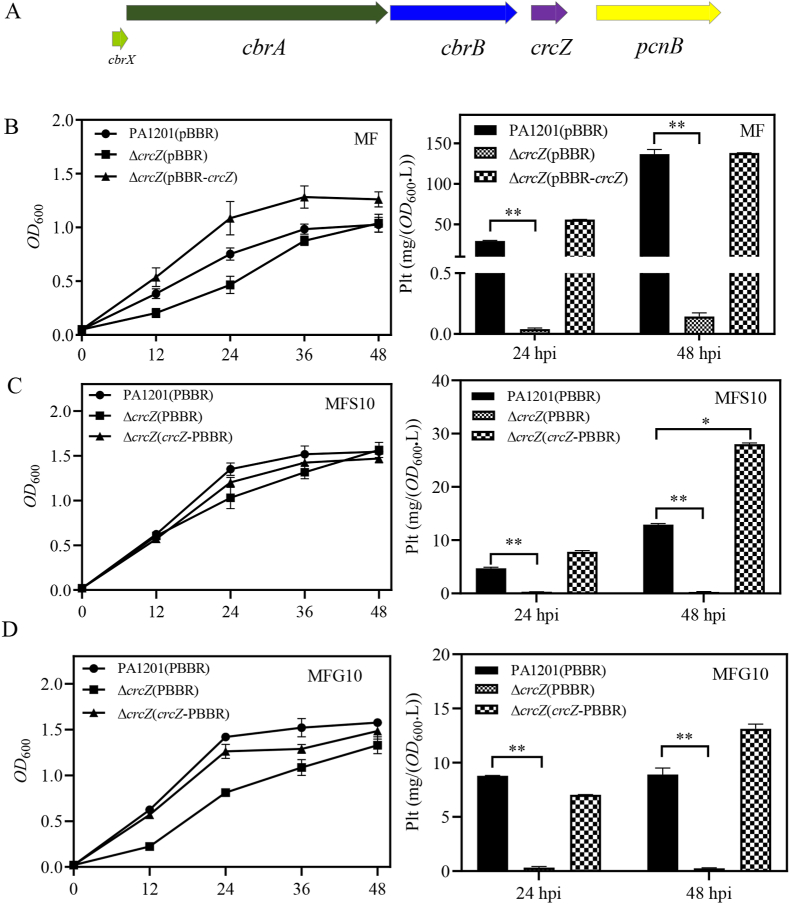
**SRNA CrcZ positively regulates Plt biosynthesis in PA1201.** (A) *cbrA*, *cbrB*, and *crcZ* loci on PA1201 chromosome. (B) Growth kinetics and Plt levels of the strains PA1201(pBBR), Δ*crcZ*(pBBR) and Δ*crcZ*(pBBR-*crcZ*) at 24- and 48-hpi in MF medium, in (C) MFS10 medium, or in (D) MFG10 medium.

The *crcZ* gene, encoding *crcZ* sRNA, is located immediately downstream of *cbrB* (Fig. 6A). It has been shown that CbrB could bind the regulatory regions of *crcZ* and activate its transcription from RpoN-dependent promoters [41]. To investigate whether *crcZ* is required for fructose-dependent Plt biosynthesis, PA1201(pBBR), Δ*crcZ*(pBBR) and Δ*crcZ*(pBBR-*crcZ*) strains were constructed and grown in MF medium. In Δ*crcZ*(pBBR) cultures, Plt level was strongly diminished at 24- and 48-hpi compared to PA1201(pBBR) cultures, whereas in Δ*crcZ*(pBBR-*crcZ*) cultures, Plt production was restored to levels obtained in PA1201(pBBR) control cultures (Fig. 6B).

In MFS10 or MFG10 medium, the Plt level produced by the Δ*crcZ*(pBBR) strain was significantly lower than that produced by PA1201(pBBR); c*rcZ* overexpression in Δ*crcZ*(pBBR-*crcZ*) increased significantly Plt level at 48 hpi in MFS10 medium, exceeding wild-type Plt level, and to a lesser extend in MFG10 medium (Fig. 6C and D). These findings suggested that *crcZ* partly mediates the antagonistic effects of glucose and succinate on fructose-induced Plt biosynthesis.

### 3.7 Hfq is involved in fructose promoting Plt biosynthesis and mediates the antagonistic effects of succinic acid and glucose on fructose-induced Plt biosynthesis

**Fig. 7:**
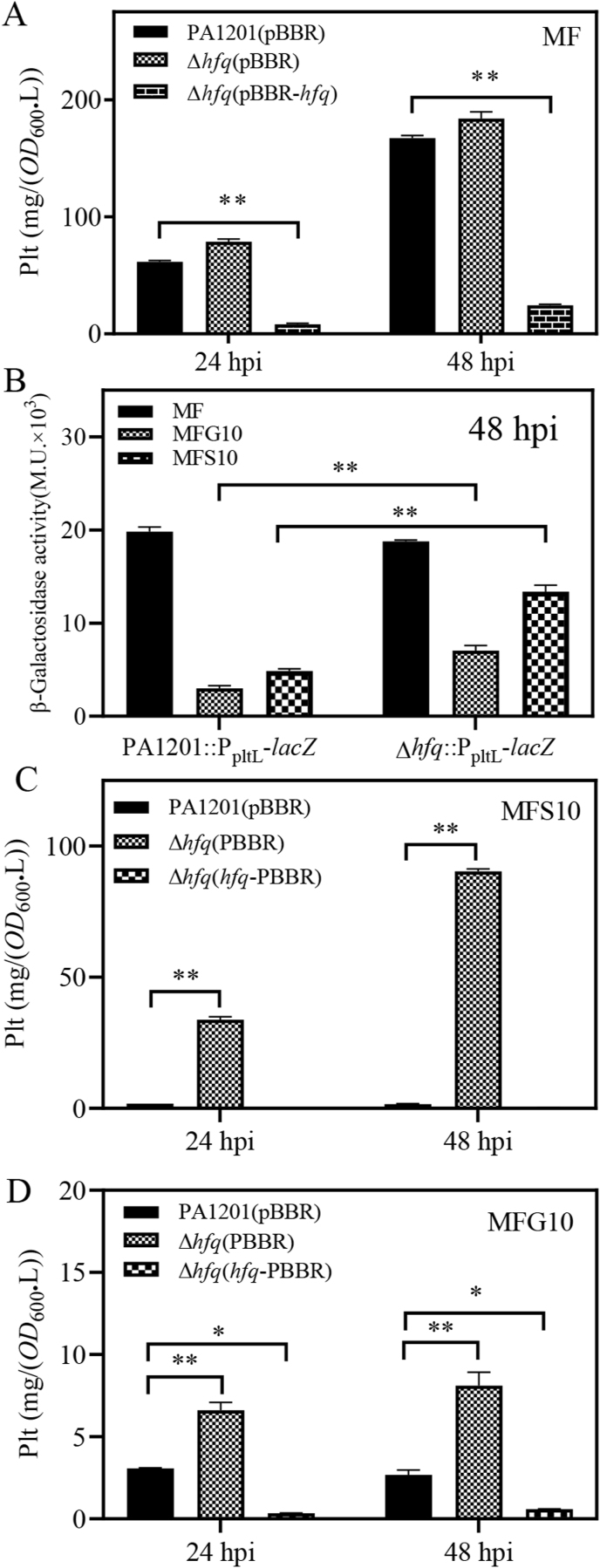
**Role of Hfq in Plt biosynthesis.** (A) Plt production by PA1201(pBBR), Δ*hfq*(pBBR), and Δ*hfq*(pBBR-*hfq*) at 24- and 48-hpi in MF medium. (B) P_pltL_-dependent β-galactosidase activity in strains PA1201 and Δ*hfq* cultured in MF, MFG10, or MFS10 medium. (C) Plt production by PA1201(pBBR), Δ*hfq*(pBBR) and Δ*hfq*(pBBR-*hfq*) at 24- and 48-hpi in MFS10 medium, or in (D) MFG10 medium.

Hfq is a pleiotropic regulator notably involved in CCR in *Pseudomonas* and related bacterial species [42]. To investigate the possible roles of *hfq* in fructose-induced Plt biosynthesis, strains deleted for *hfq* (Δ*hfq*) or overexpressing *hfq* [Δ*hfq*(pBBR-*hfq*)] were generated in PA1201 and grown in MF medium. Deletion of *hfq* had no significant impact on Plt level at 24- or 48-hpi (Fig. 7A). Consistently, *pltL* promoter-dependent β-galactosidase activity in the reporter strain PA1201:P_pltL_-*lacZ* at 48 hpi was not different from that in Δ*hfq*:P_pltL_-*lacZ* (Fig. 7B). However, overexpression of *hfq* in Δ*hfq*(pBBR-*hfq*) reduced Plt biosynthesis to a level much lower than that in wild-type PA1201 (Fig. 7A), indicating an inhibitory effect of Hfq on fructose-induced Plt synthesis.

In MFS10 medium, Plt production by the Δ*hfq* mutant reached 39.5 mg/(OD_600_.L) at 48 hpi, which was significantly higher than the production achieved by wild-type PA1201 [8.4 mg/(OD_600_.L); Fig. 7C]. This result indicated that the inhibition of fructose-induced Plt synthesis by succinic acid required Hfq. Overexpression of *hfq* in Δ*hfq* restored Plt inhibition to wild-type level (Fig. 7C). Consistently, P_pltL_-dependent β-galactosidase activity in the reporter strain Δ*hfq*:P_pltL_-*lacZ* was significantly higher than that in PA1201:P_pltL_-*lacZ* when cultured in MFS10 (Fig. 7B). Similar trends in Plt level and P_pltL_-dependent β-galactosidase activity were observed in MFG10 medium (Fig. 7B–D). These findings suggest that Hfq mediates the antagonistic effects of succinate and glucose on fructose-dependent Plt biosynthesis.

### 3.8 Crc protein is involved in fructose promoting Plt biosynthesis and mediates the antagonistic effects of succinic acid and glucose on fructose-induced Plt biosynthesis

**Fig. 8:**
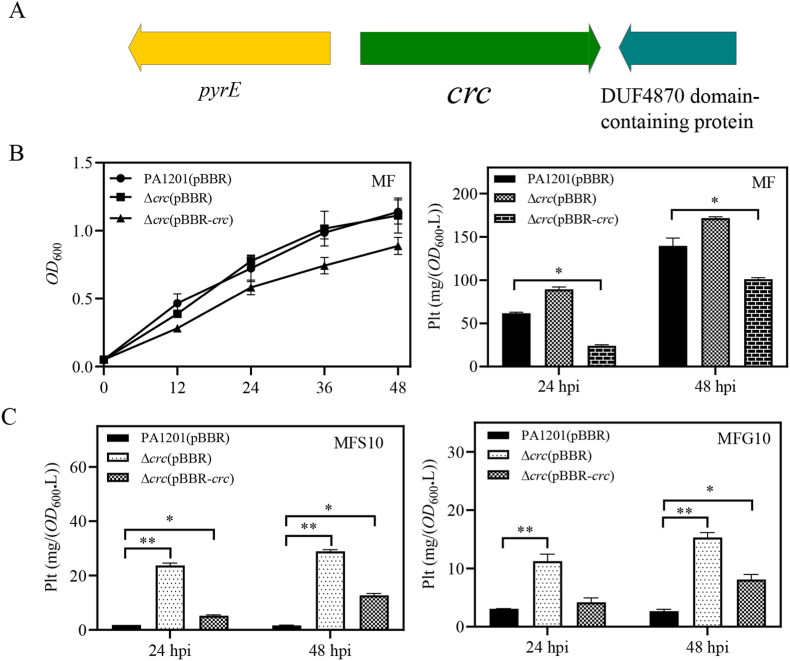
**Role of Crc in Plt biosynthesis.** (A) *crc* locus on PA1201 chromosome. (B)Growth kinetics and Plt concentration in PA1201(pBBR), Δ*crc*(pBBR), and Δ*crc*(pBBR-*crc*) at 24- and 48-hpi in MF medium, or in (C) MFS10 and MFG10 media.

The Crc protein can stabilize Hfq binding to the A-rich motifs of target mRNAs to form tripartite Hfq–RNA–Crc complexes [43]. In PA1201, the 780-bp *crc* gene is flanked by the *pyrE* gene, encoding an orotate phosphoribosyltransferase, and the gene encoding DUF4870 domain-containing protein (Fig. 8A). To investigate the possible role of Crc in fructose-promoted Plt biosynthesis, strains deleted for *crc* [Δ*crc*(pBBR)] or overexpressing *crc* [Δ*crc*(pBBR-*crc*)] were constructed in PA1201 and grown in MF. Plt levels in Δ*crc* was not significantly different from that in PA1201 at 24 and 48 hpi (Fig. 8B). In contrast, overexpression of *crc* in Δ*crc*(pBBR-*crc*) decreased Plt production below wild-type level. Further, no additive effect was observed on Plt biosynthesis in the double knockout strain Δ*hfq*Δ*crc*, which suggested that these two gene products acted in the same inhibitory pathway (Fig. S3).

In MFS10 or MFG10 medium, the Plt levels obtained with Δ*crc* at 48 hpi, which were respectively, 15.4 and 20.7 mg/(OD_600_.L), were significantly higher than that obtained with PA1201 [2.7 and 7.0 mg/(OD_600_.L), respectively]. *Crc* overexpression in Δ*crc*(pBBR*-crc*) strain restored the inhibition of Plt production observed in wild-type PA1201 cultured in MFG10 and MFS10 media (Fig. 8C). Thus, the antagonizing effect of glucose or succinate on fructose-dependent Plt biosynthesis is mediated by Crc.

## 4 Discussion

The broad-spectrum antimicrobial property makes Plt a promising candidate for the development of new biopesticides. However, Plt yield in wild-type *Pseudomonas* strains is far too low to meet industrial demands. The type of carbon source and its availability was shown to affect the production of bacterial antimicrobials in various bacterial genera [44]. The environment and nutrients have been identified as influential factors for Plt production in *Pseudomonas*. For example, the co-production of approximately 150 mg/L of Plt and 500 mg/L of 2,4-diacetylphloroglucinol, another antimicrobial metabolite, was achieved by flask cultivation in a medium containing approximately 2% ethanol [24]. Duffy et al. found that Plt production was stimulated by Zn^2+^, Co^2+^, and glycerol, but repressed by glucose; adding glucose to NBY medium could inhibit Plt production by *Pseudomonas* fluorescens CHA0, while CHA0 produced more Plt using mannitol and glycerol as sole carbon sources [23]. All these findings suggest that the environment- and nutrients-dependent regulation of Plt biosynthesis deserves further research. In this study, we systematically investigated the effects of 11 carbon sources on bacterial growth and Plt production in PA1201. Our results showed that the nutrient-poor MM medium favored Plt production. Addition of fructose, mannitol, or glycerol promoted Plt biosynthesis, whereas addition of glucose or succinic acid enhanced bacterial growth but decreased the Plt production. These results are generally consistent with the previous finding. However, the present study further showed that fructose is more efficient than mannitol and glycerol in Plt promotion (Fig. 2B). Based on these results, we developed the fructose-containing medium MF and obtained a Plt yield of 190.26 mg/L in wild-type PA1201. This is the highest Plt titer and yield ever obtained so far.

CCR is a general mechanism that facilitates the catabolism (assimilation) of carbon from different sources, supports efficient growth, and represses the catabolism of other potentially useable carbon sources that are less energetically efficient [45]. Thus, CCR allows bacterial cells to preferentially assimilate a single carbon compound among multiple carbon sources. In addition, CCR potentially control antibiotic biosynthesis indirectly in *Pseudomonas* spp [46]. The CCR regulatory cascade is composed of three layers: the two-component system CbrA/CbrB, the CrcZ/Y sRNAs, and the translational repressor Crc [30]. In this study, glucose or succinic acid was shown to promote PA1201 growth, but antagonized fructose-dependent Plt promotion (Fig. 3). These results suggest that CCR controls the utilization of carbon sources and Plt production in PA1201. Further, fructose was shown to promote Plt production directly by increasing the transcription of *pltL* operon. CCR-associated regulators are required for fructose-dependent *pltL* expression and Plt production (Fig. 5, Fig. 6, Fig. 7, Fig. 8B). Furthermore, CCR-associated regulators are also required for the glucose- or succinic acid-dependent antagonization of fructose production (Fig. 5BCE, 6CD, 7CD, 8C). The molecular mechanisms underlying the regulation of *pltL* expression by CCR in PA1201 remain to be understood. A more complete understanding of these mechanisms can help optimizing Plt production and its industrial application.

**Fig. 9:**
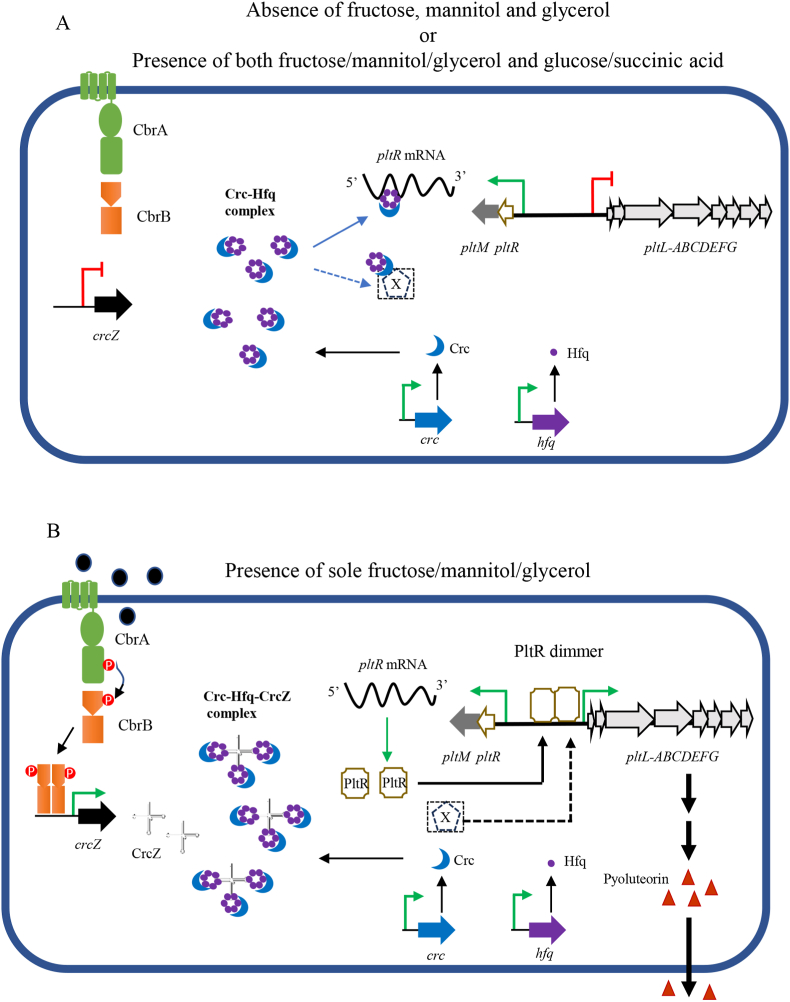
**Model of regulation of pyoluteorin biosynthesis by carbohydrates via carbon catabolite repression (CCR) mechanism.** (A) Plt production is limited in the absence of fructose, mannitol and glycerol or in the presence of both fructose/mannitol/glycerol and glucose/succinic acid. (B) Plt production is induced in the presence of sole fructose/mannitol/glycerol. The dashed line indicates an alternative pathway to activate *pltL* transcription via the not yet identified regulator X.

The two-component CbrA/CbrB system is involved in nutritional adaptation and was first described in *P. aeruginosa* as a regulator of hierarchical utilization of various carbon sources [29]. To date, no orthologous system has been described in other species, and its activating signals remain elusive, although some authors suggested that it could include the C:N balance [40]. In this study, we found that at least three carbohydrates, fructose, mannitol, and glycerol, could promote Plt production. Glucose or succinic acid antagonized fructose-dependent Plt production. Thus, these carbohydrates are unlikely the direct activators of the CbrA/CbrB system and further investigation is necessary to clarify the underlying mechanisms of this regulation. From the current results, we proposed a working model to explain how different carbohydrates affect Plt production in PA1201 cells (Fig. 9). In absence of fructose, mannitol, or glycerol, or in presence of both fructose and glucose or fructose and succinic acid, the CbrA/CbrB system is not activated, no sRNA CrcZ is expressed, and Hfq and Crc form a two-protein complex [30,43]. This complex binds *pltR* mRNA, inhibiting PltR protein production, thereby impeding the initiation of *pltL* expression [47]. Nonetheless, we should not exclude the possibility that the Crc:Hfq complex binds an not yet identified X regulator that activates the transcription of *pltL* operon (Fig. 9A). In sole presence of fructose, mannitol or glycerol, the CbrA/CbrB system is activated and phosphorylated CbrB binds *crcZ* promoter to initiate the transcription of *CrcZ* sRNA [27]. Hfq and Crc proteins bind *CrcZ* to form a three-partite complex [48,49]. This complex loses the capacity to bind *pltR* mRNA or the regulator X, enabling PltR protein translation. PltR dimers activates the promoter of *pltL* operon, which in turn, initiates Plt biosynthesis [10] (Fig. 9B).

In summary, this study identified the optimal medium and carbohydrate for Plt biosynthesis. Fructose promotes Plt biosynthesis through the *pltL* cluster via a CCR mechanism involving the CbrAB-CrcZ-Hfq/Crc pathway. Glucose or succinic acid antagonizes the fructose-dependent Plt induction. These findings provide new insight into Plt biosynthesis regulation and new clues to genetically modify *Pseudomonas* for high yield of Plt. Future work is required to determine how fructose induces *pltL* expression and consequently induces Plt biosynthesis.

## CRediT authorship contribution statement

**Ying Cui:** Methodology, Investigation, Writing – original draft. **Kai Song:** Data curation, Formal analysis. **Zi-Jing Jin:** Conceptualization, Methodology. **Learn-Han Lee:** Writing – review & editing. **Chitti Thawai:** Data curation, Conceptualization. **Ya-Wen He:** Supervision, Writing – review & editing, Funding acquisition, Project administration.

## Declaration of competing interest

The authors have no interests to declare.
