# Supplementary material for: Fructose promotes pyoluteorin biosynthesis via the CbrAB-CrcZ-Hfq/Crc pathway in the biocontrol strain Pseudomonas PA1201
Source: Synth Syst Biotechnol. 2023 Sep 21;8(4):618–28. doi: 10.1016/j.synbio.2023.09.004 (PMC10562864; doi:10.1016/j.synbio.2023.09.004)
Supplement: Multimedia component 1 [file mmc1.pptx]

## Slide 1
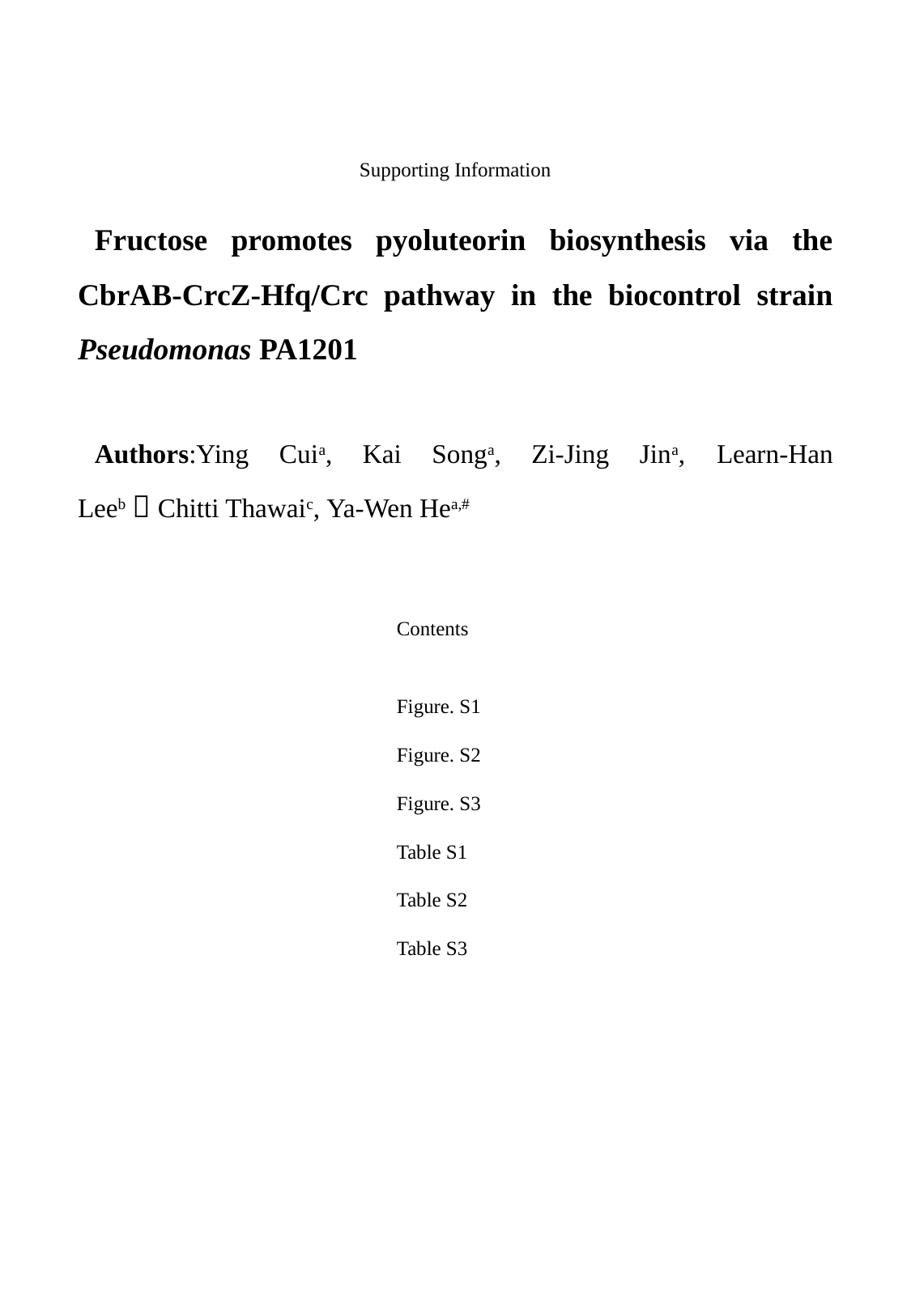

Supporting Information
Fructose promotes pyoluteorin biosynthesis via the CbrAB-CrcZ-Hfq/Crc pathway in the biocontrol strain Pseudomonas PA1201
Authors:Ying Cuia, Kai Songa, Zi-Jing Jina, Learn-Han Leeb，Chitti Thawaic, Ya-Wen Hea,#
Contents
Figure. S1
Figure. S2
Figure. S3
Table S1
Table S2
Table S3

## Slide 2
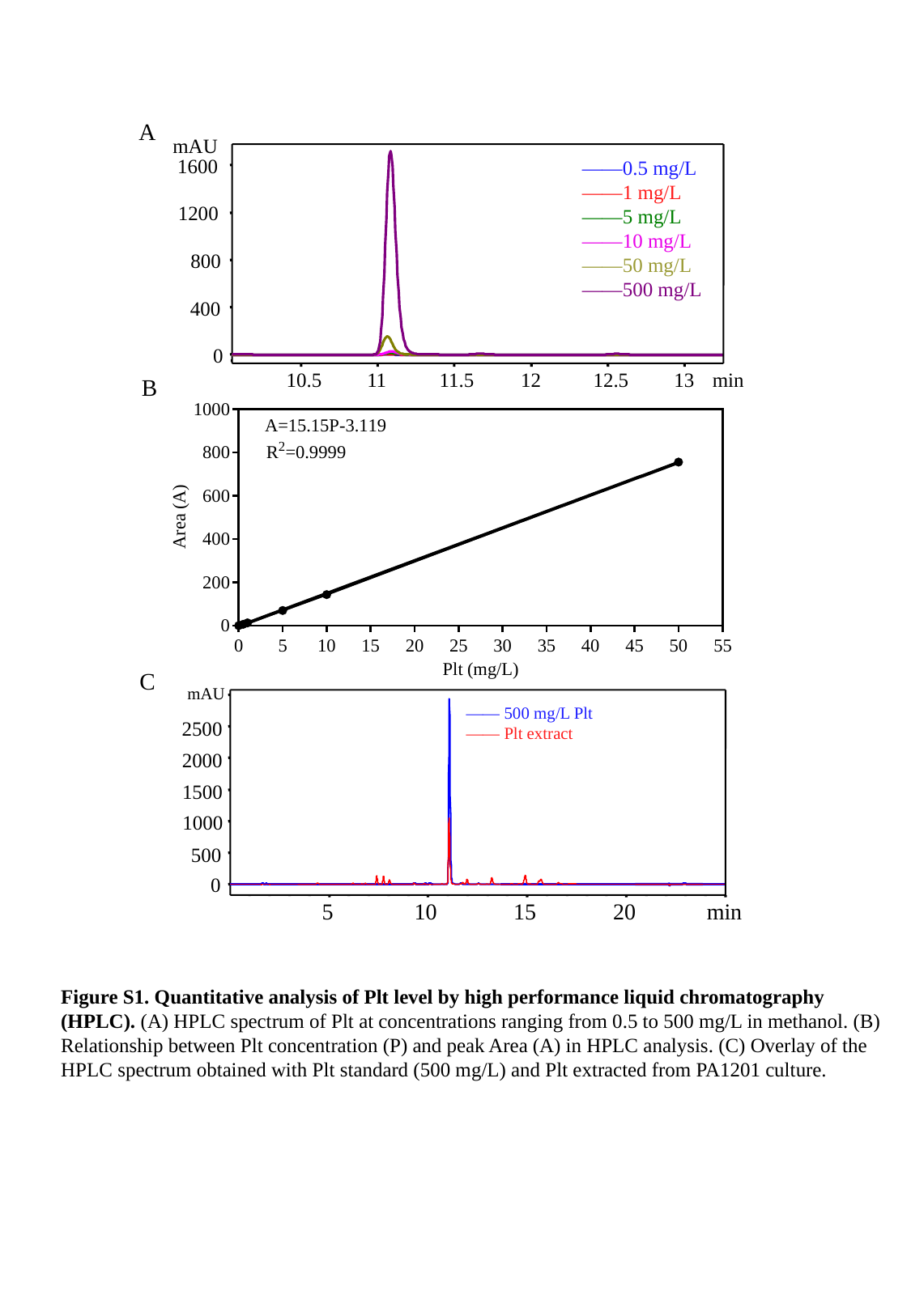

A
mAU
——0.5 mg/L
——1 mg/L
——5 mg/L
——10 mg/L
——50 mg/L
——500 mg/L
1600
1200
800
400
0
10.5
11
11.5
12
12.5
13
min
B
C
mAU
—— 500 mg/L Plt
—— Plt extract
2500
2000
1500
1000
500
0
5
10
15
20
min
Figure S1. Quantitative analysis of Plt level by high performance liquid chromatography (HPLC). (A) HPLC spectrum of Plt at concentrations ranging from 0.5 to 500 mg/L in methanol. (B) Relationship between Plt concentration (P) and peak Area (A) in HPLC analysis. (C) Overlay of the HPLC spectrum obtained with Plt standard (500 mg/L) and Plt extracted from PA1201 culture.

## Slide 3
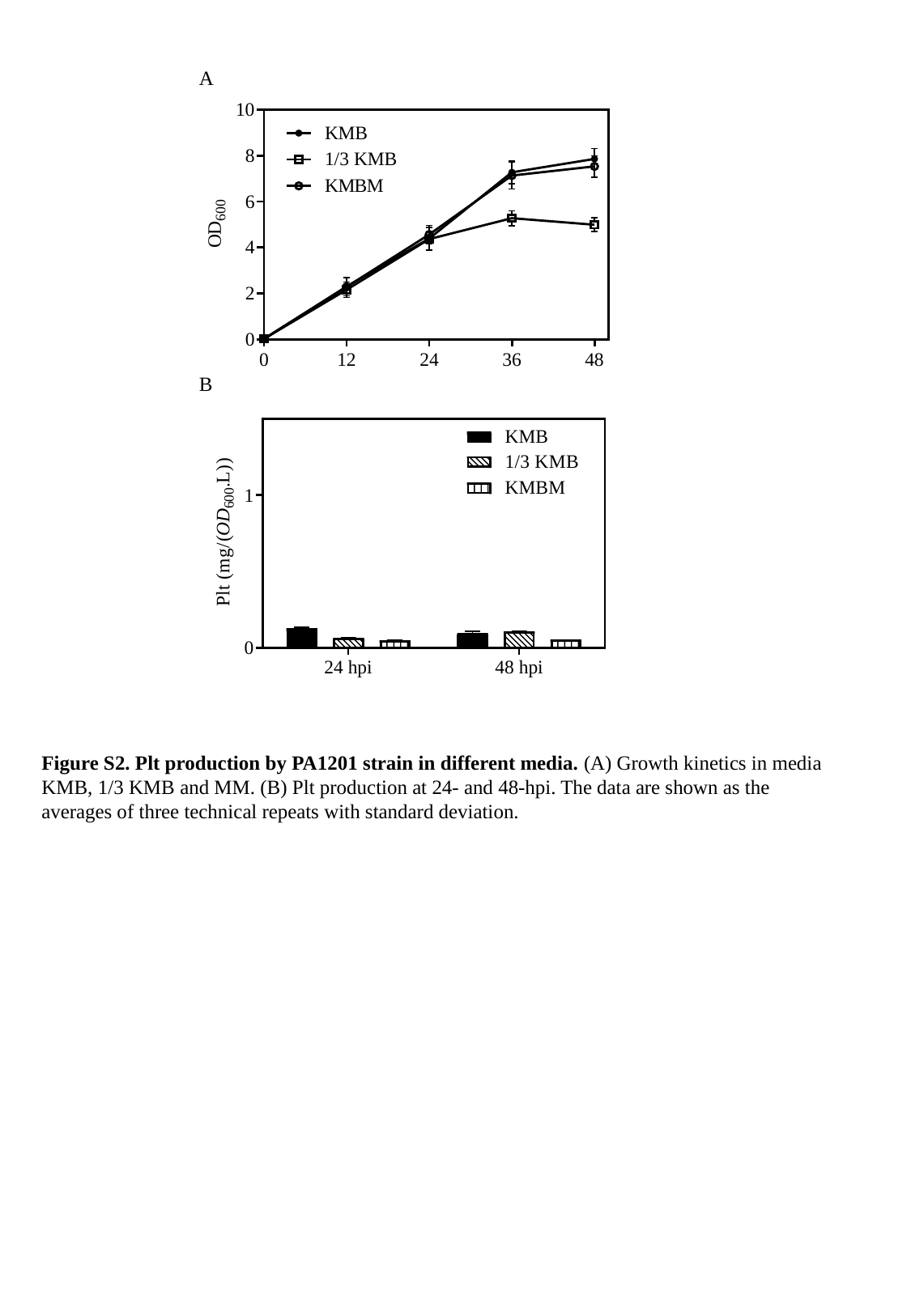

A
B
Figure S2. Plt production by PA1201 strain in different media. (A) Growth kinetics in media KMB, 1/3 KMB and MM. (B) Plt production at 24- and 48-hpi. The data are shown as the averages of three technical repeats with standard deviation.

## Slide 4
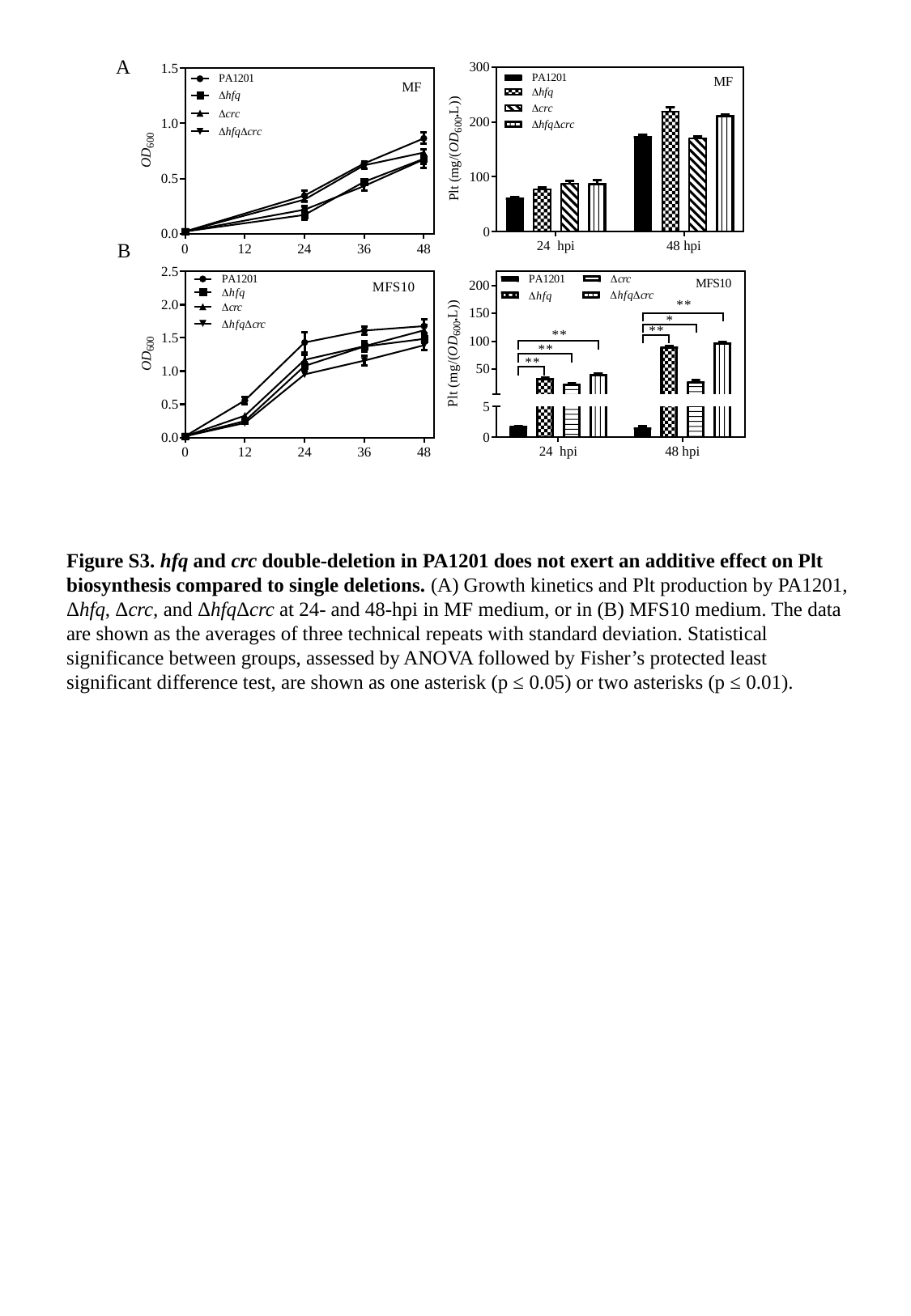

A
B
Figure S3. hfq and crc double-deletion in PA1201 does not exert an additive effect on Plt biosynthesis compared to single deletions. (A) Growth kinetics and Plt production by PA1201, Δhfq, Δcrc, and ΔhfqΔcrc at 24- and 48-hpi in MF medium, or in (B) MFS10 medium. The data are shown as the averages of three technical repeats with standard deviation. Statistical significance between groups, assessed by ANOVA followed by Fisher’s protected least significant difference test, are shown as one asterisk (p ≤ 0.05) or two asterisks (p ≤ 0.01).

## Slide 5
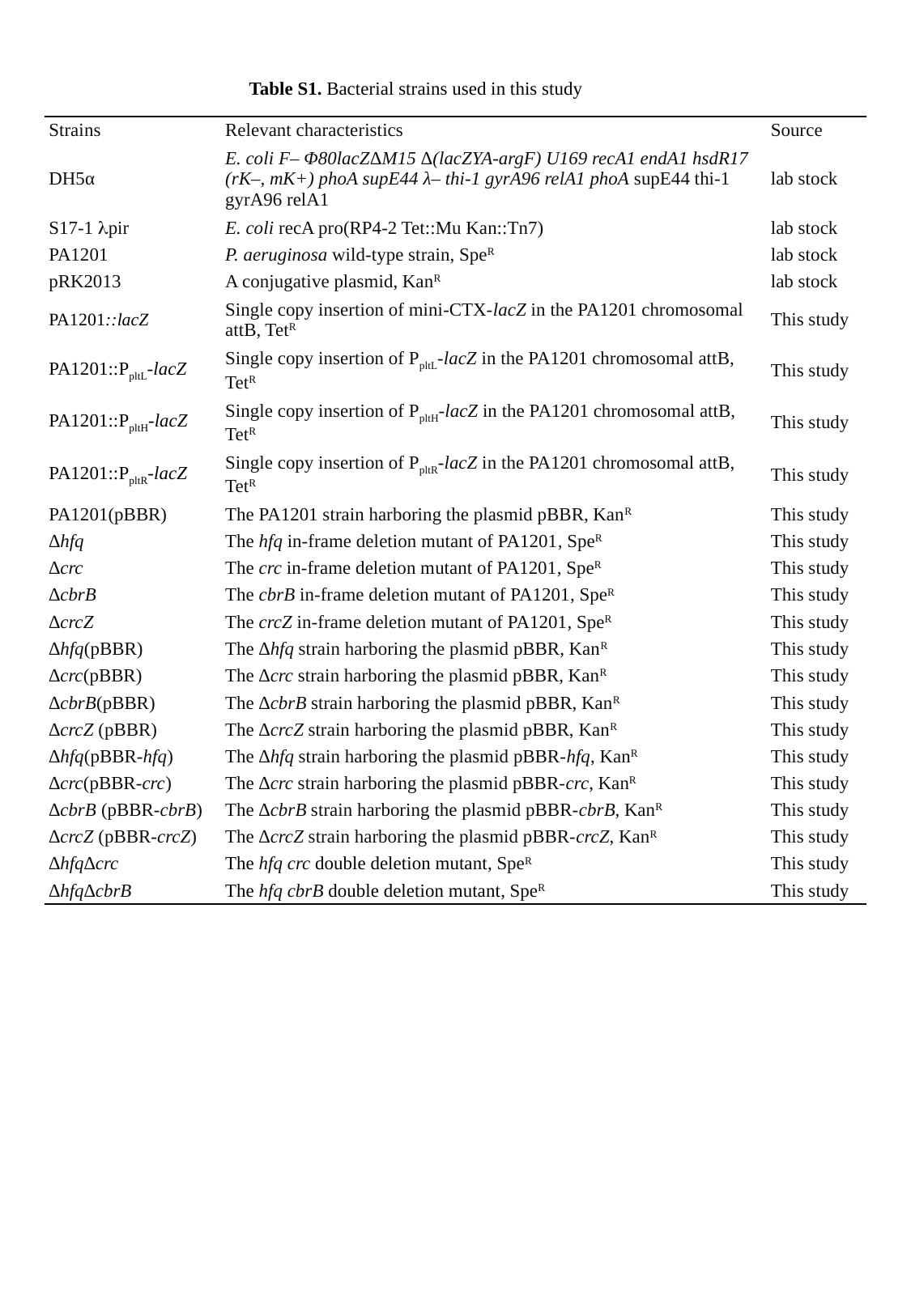

Table S1. Bacterial strains used in this study
| Strains | Relevant characteristics | Source |
| --- | --- | --- |
| DH5α | E. coli F– Φ80lacZΔM15 Δ(lacZYA-argF) U169 recA1 endA1 hsdR17 (rK–, mK+) phoA supE44 λ– thi-1 gyrA96 relA1 phoA supE44 thi-1 gyrA96 relA1 | lab stock |
| S17-1 pir | E. coli recA pro(RP4-2 Tet::Mu Kan::Tn7) | lab stock |
| PA1201 | P. aeruginosa wild-type strain, SpeR | lab stock |
| pRK2013 | A conjugative plasmid, KanR | lab stock |
| PA1201::lacZ | Single copy insertion of mini-CTX-lacZ in the PA1201 chromosomal attB, TetR | This study |
| PA1201::PpltL-lacZ | Single copy insertion of PpltL-lacZ in the PA1201 chromosomal attB, TetR | This study |
| PA1201::PpltH-lacZ | Single copy insertion of PpltH-lacZ in the PA1201 chromosomal attB, TetR | This study |
| PA1201::PpltR-lacZ | Single copy insertion of PpltR-lacZ in the PA1201 chromosomal attB, TetR | This study |
| PA1201(pBBR) | The PA1201 strain harboring the plasmid pBBR, KanR | This study |
| ∆hfq | The hfq in-frame deletion mutant of PA1201, SpeR | This study |
| ∆crc | The crc in-frame deletion mutant of PA1201, SpeR | This study |
| ∆cbrB | The cbrB in-frame deletion mutant of PA1201, SpeR | This study |
| ∆crcZ | The crcZ in-frame deletion mutant of PA1201, SpeR | This study |
| ∆hfq(pBBR) | The ∆hfq strain harboring the plasmid pBBR, KanR | This study |
| ∆crc(pBBR) | The ∆crc strain harboring the plasmid pBBR, KanR | This study |
| ∆cbrB(pBBR) | The ∆cbrB strain harboring the plasmid pBBR, KanR | This study |
| ∆crcZ (pBBR) | The ∆crcZ strain harboring the plasmid pBBR, KanR | This study |
| ∆hfq(pBBR-hfq) | The ∆hfq strain harboring the plasmid pBBR-hfq, KanR | This study |
| ∆crc(pBBR-crc) | The ∆crc strain harboring the plasmid pBBR-crc, KanR | This study |
| ∆cbrB (pBBR-cbrB) | The ∆cbrB strain harboring the plasmid pBBR-cbrB, KanR | This study |
| ∆crcZ (pBBR-crcZ) | The ∆crcZ strain harboring the plasmid pBBR-crcZ, KanR | This study |
| ∆hfq∆crc | The hfq crc double deletion mutant, SpeR | This study |
| ∆hfq∆cbrB | The hfq cbrB double deletion mutant, SpeR | This study |
#

## Slide 6
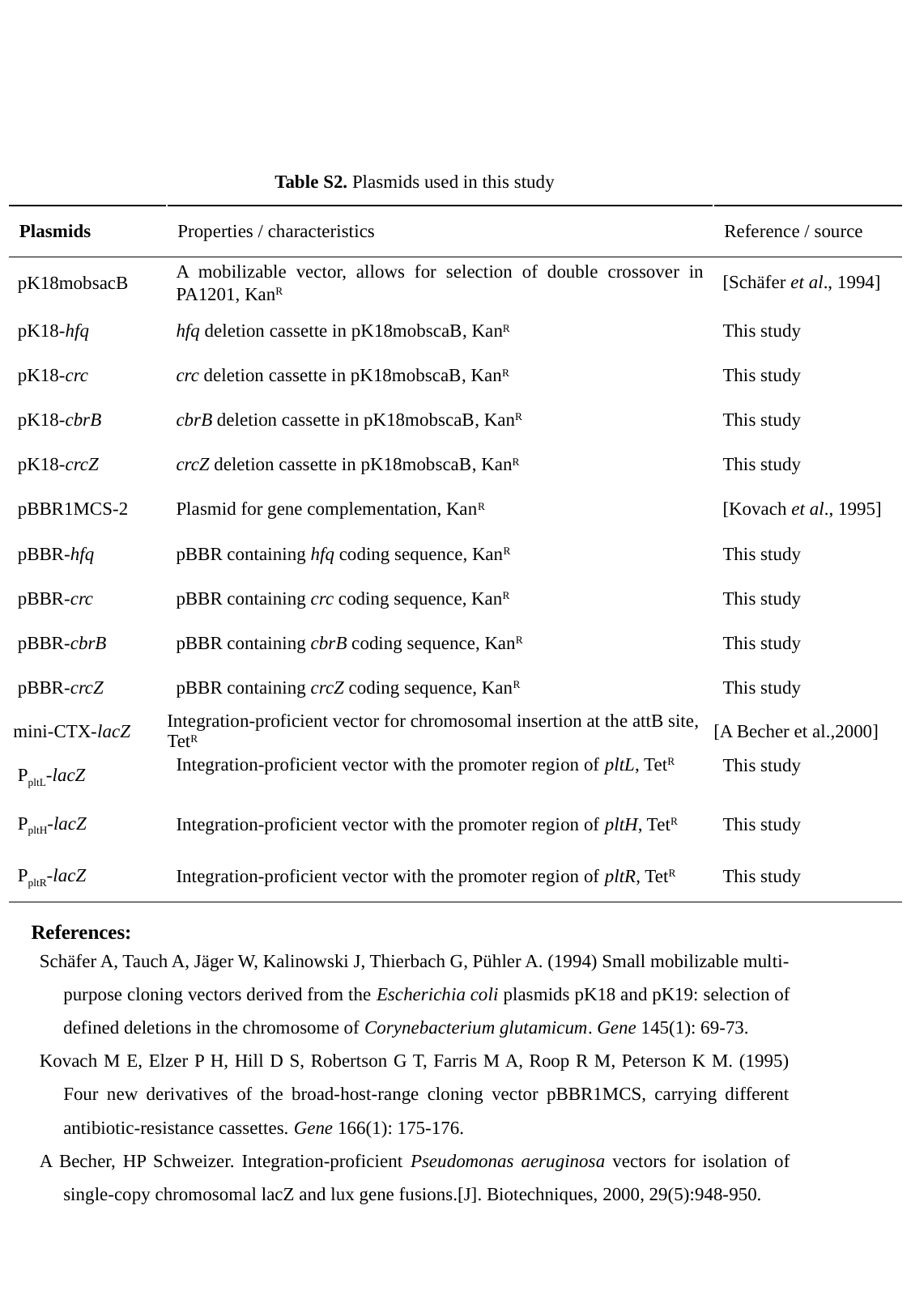

Table S2. Plasmids used in this study
| Plasmids | Properties / characteristics | Reference / source |
| --- | --- | --- |
| pK18mobsacB | A mobilizable vector, allows for selection of double crossover in PA1201, KanR | [Schäfer et al., 1994] |
| pK18-hfq | hfq deletion cassette in pK18mobscaB, KanR | This study |
| pK18-crc | crc deletion cassette in pK18mobscaB, KanR | This study |
| pK18-cbrB | cbrB deletion cassette in pK18mobscaB, KanR | This study |
| pK18-crcZ | crcZ deletion cassette in pK18mobscaB, KanR | This study |
| pBBR1MCS-2 | Plasmid for gene complementation, KanR | [Kovach et al., 1995] |
| pBBR-hfq | pBBR containing hfq coding sequence, KanR | This study |
| pBBR-crc | pBBR containing crc coding sequence, KanR | This study |
| pBBR-cbrB | pBBR containing cbrB coding sequence, KanR | This study |
| pBBR-crcZ | pBBR containing crcZ coding sequence, KanR | This study |
| mini-CTX-lacZ | Integration-proficient vector for chromosomal insertion at the attB site, TetR | [A Becher et al.,2000] |
| PpltL-lacZ | Integration-proficient vector with the promoter region of pltL, TetR | This study |
| PpltH-lacZ | Integration-proficient vector with the promoter region of pltH, TetR | This study |
| PpltR-lacZ | Integration-proficient vector with the promoter region of pltR, TetR | This study |
References:
Schäfer A, Tauch A, Jäger W, Kalinowski J, Thierbach G, Pühler A. (1994) Small mobilizable multi-purpose cloning vectors derived from the Escherichia coli plasmids pK18 and pK19: selection of defined deletions in the chromosome of Corynebacterium glutamicum. Gene 145(1): 69-73.
Kovach M E, Elzer P H, Hill D S, Robertson G T, Farris M A, Roop R M, Peterson K M. (1995) Four new derivatives of the broad-host-range cloning vector pBBR1MCS, carrying different antibiotic-resistance cassettes. Gene 166(1): 175-176.
A Becher, HP Schweizer. Integration-proficient Pseudomonas aeruginosa vectors for isolation of single-copy chromosomal lacZ and lux gene fusions.[J]. Biotechniques, 2000, 29(5):948-950.

## Slide 7
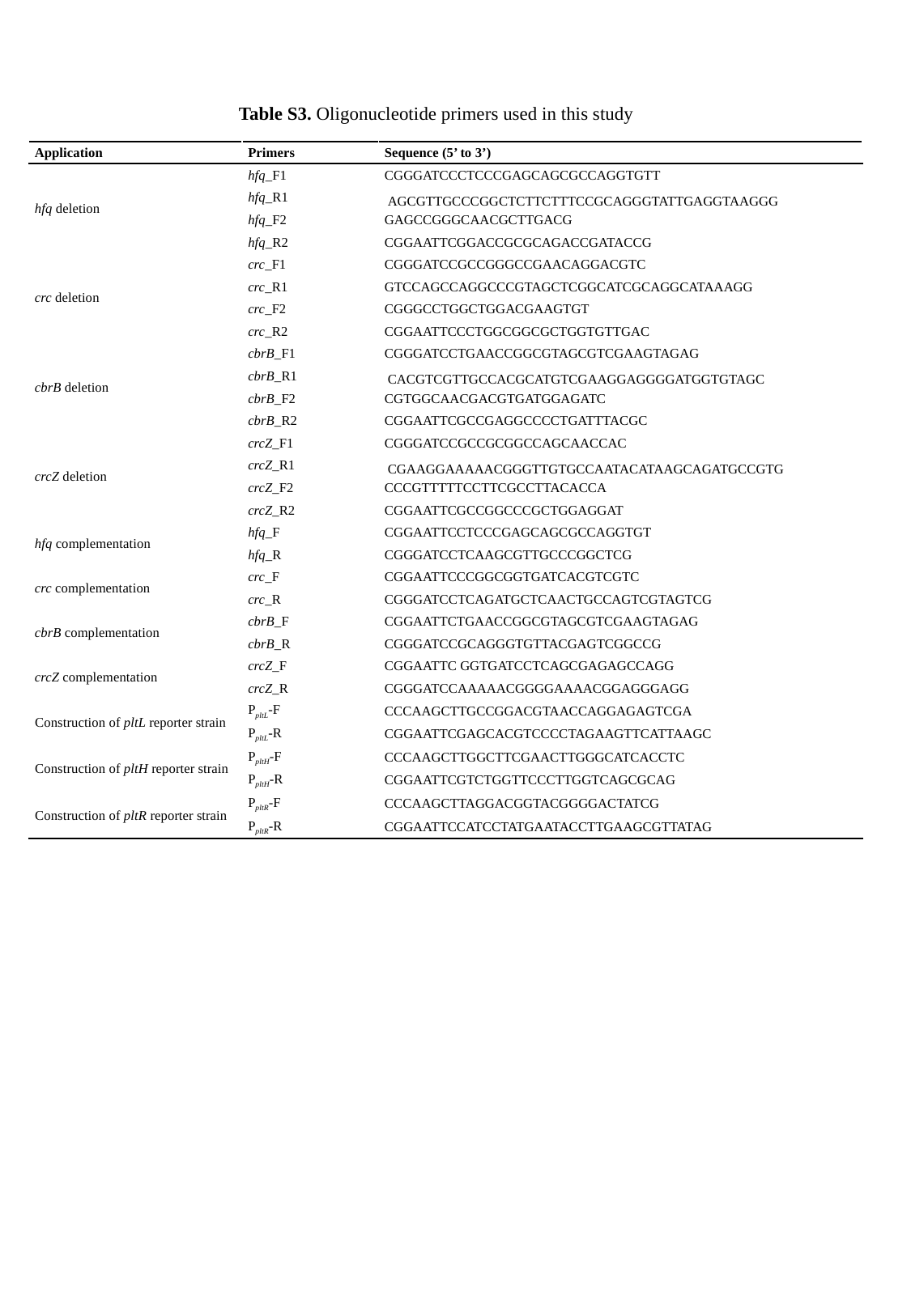

Table S3. Oligonucleotide primers used in this study
| Application | Primers | Sequence (5’ to 3’) |
| --- | --- | --- |
| hfq deletion | hfq\_F1 | CGGGATCCCTCCCGAGCAGCGCCAGGTGTT |
| | hfq\_R1 | AGCGTTGCCCGGCTCTTCTTTCCGCAGGGTATTGAGGTAAGGG |
| | hfq\_F2 | GAGCCGGGCAACGCTTGACG |
| | hfq\_R2 | CGGAATTCGGACCGCGCAGACCGATACCG |
| crc deletion | crc\_F1 | CGGGATCCGCCGGGCCGAACAGGACGTC |
| | crc\_R1 | GTCCAGCCAGGCCCGTAGCTCGGCATCGCAGGCATAAAGG |
| | crc\_F2 | CGGGCCTGGCTGGACGAAGTGT |
| | crc\_R2 | CGGAATTCCCTGGCGGCGCTGGTGTTGAC |
| cbrB deletion | cbrB\_F1 | CGGGATCCTGAACCGGCGTAGCGTCGAAGTAGAG |
| | cbrB\_R1 | CACGTCGTTGCCACGCATGTCGAAGGAGGGGATGGTGTAGC |
| | cbrB\_F2 | CGTGGCAACGACGTGATGGAGATC |
| | cbrB\_R2 | CGGAATTCGCCGAGGCCCCTGATTTACGC |
| crcZ deletion | crcZ\_F1 | CGGGATCCGCCGCGGCCAGCAACCAC |
| | crcZ\_R1 | CGAAGGAAAAACGGGTTGTGCCAATACATAAGCAGATGCCGTG |
| | crcZ\_F2 | CCCGTTTTTCCTTCGCCTTACACCA |
| | crcZ\_R2 | CGGAATTCGCCGGCCCGCTGGAGGAT |
| hfq complementation | hfq\_F | CGGAATTCCTCCCGAGCAGCGCCAGGTGT |
| | hfq\_R | CGGGATCCTCAAGCGTTGCCCGGCTCG |
| crc complementation | crc\_F | CGGAATTCCCGGCGGTGATCACGTCGTC |
| | crc\_R | CGGGATCCTCAGATGCTCAACTGCCAGTCGTAGTCG |
| cbrB complementation | cbrB\_F | CGGAATTCTGAACCGGCGTAGCGTCGAAGTAGAG |
| | cbrB\_R | CGGGATCCGCAGGGTGTTACGAGTCGGCCG |
| crcZ complementation | crcZ\_F | CGGAATTC GGTGATCCTCAGCGAGAGCCAGG |
| | crcZ\_R | CGGGATCCAAAAACGGGGAAAACGGAGGGAGG |
| Construction of pltL reporter strain | PpltL-F | CCCAAGCTTGCCGGACGTAACCAGGAGAGTCGA |
| | PpltL-R | CGGAATTCGAGCACGTCCCCTAGAAGTTCATTAAGC |
| Construction of pltH reporter strain | PpltH-F | CCCAAGCTTGGCTTCGAACTTGGGCATCACCTC |
| | PpltH-R | CGGAATTCGTCTGGTTCCCTTGGTCAGCGCAG |
| Construction of pltR reporter strain | PpltR-F | CCCAAGCTTAGGACGGTACGGGGACTATCG |
| | PpltR-R | CGGAATTCCATCCTATGAATACCTTGAAGCGTTATAG |
